# NLRX1 contributes to immunotherapeutic resistance of head and neck squamous cell carcinoma by suppressing type I interferon pathway

**DOI:** 10.3389/fimmu.2026.1890178

**Published:** 2026-08-19

**Authors:** Yuqi Wu, Yuchen Jiang, Dan Pan, Zhenyu Zhang, Qianming Chen, Xiaobo Luo

**Affiliations:** State Key Laboratory of Oral Diseases, National Clinical Research Center for Oral Diseases, Chinese Academy of Medical Sciences Research Unit of Oral Carcinogenesis and Management, West China Hospital of Stomatology, Sichuan University, Chengdu, China

**Keywords:** HNSCC, immunotherapy, NLRX1, PD-L1, STING

## Abstract

**Background:**

Head and neck squamous cell carcinoma (HNSCC) faces challenges with limited immune checkpoint inhibitors (ICIs) efficacy. This study identifies NLRX1, highly expressed in HNSCC tumor cells, as a key driver of immune evasion and ICIs’ resistance.

**Methods:**

This study investigated the role of NLRX1 in HNSCC immune evasion and ICIs resistance. We performed retrospective analyses on patient cohorts to correlate NLRX1 expression with clinical outcomes. Bioinformatic analyses and immunofluorescence staining of human HNSCC tissues were employed to assess associations between immune cell infiltration, gene expression correlations and PD-L1 levels on antigen-presenting cells (APCs). *In vitro* experiments were conducted to investigate the regulatory role of NLRX1 on cell proliferation and gene expression profiling by overexpressing or knocking down NLRX1. *In vivo* studies of subcutaneous tumor models in both immunodeficient and immunocompetent mice were utilized to assess the function of NLRX1 on tumor progression.

**Results:**

The expression NLRX1 is correlated with unfavorable patient outcomes. NLRX1 promotes tumor progression by modulating the immune microenvironment, not directly affecting tumor cell proliferation. Mechanistically, NLRX1 suppresses the type I interferon (IFN-I) pathway by inhibiting STING expression in HNSCC. Notably, tumor cell-intrinsic NLRX1 negatively correlates with PD-L1 expression on APCs in HNSCC. *In vivo*, NLRX1 knockdown activated STING-IFN-I signaling, upregulated APC PD-L1, suppressed tumor growth, and significantly boosted anti-PD-L1 therapy in murine tumor models.

**Conclusions:**

NLRX1 mediates immune evasion in HNSCC by downregulating STING protein abundance, suppressing the IFN-I pathway, and decreasing PD-L1 expression of APCs. Targeting NLRX1 offers a promising strategy to overcome ICI resistance and improve immunotherapeutic efficacy in HNSCC.

## 1 Introduction

Head and neck squamous cell carcinoma (HNSCC) ranks among the most prevalent malignancies globally, accounting for approximately 4.6% of all cancer diagnoses (1, 2). Despite continuous advancements in therapeutic modalities such as surgery, radiotherapy, and chemotherapy, the 5-year survival rate for HNSCC patients remains below 50% (3). Besides, traditional surgical interventions often lead to severe maxillofacial deformities and functional impairments, significantly reducing patients’ quality of life. Consequently, there is an urgent need to identify novel alternative therapeutic strategies for HNSCC.

In recent years, immunotherapy has emerged as a novel promising approach for cancer management. CD8+ T cells serve as the predominant immune subset for specific tumor killing; however, tumors evolve with some mechanisms to evade the immune surveillance; for instance, tumor cells express programmed cell death protein 1 ligand 1 (PD-L1) on their surface, thus binding to programmed cell death protein 1 (PD-1) on T cells, generating inhibitory signals that impede the proliferation, activation, and function of effector T cells, thereby leading to tumor immune evasion (4, 5). Currently, immune checkpoint inhibitors (ICIs), represented by monoclonal antibodies (mAbs) targeting PD-1 or PD-L1, have achieved remarkable success in managing various malignancies, including melanoma and non-small cell lung cancer. Pembrolizumab became the first PD-1 mAb approved by the U.S. Food and Drug Administration (FDA) in 2016 for the treatment of recurrent or metastatic HNSCC (6), followed by subsequent approvals of Nivolumab and Durvalumab. However, only 15-20% of HNSCC patients have received clinical benefits upon ICIs therapy (7, 8). Therefore, the critical mechanism by which HNSCC presents poor response to ICIs remains to be elucidated, thus improving the efficacy of tumor immunotherapy (9).

Notably, recent studies indicate that the expression level of PD-L1 on the surface of antigen-presenting cells (APCs) plays a pivotal and decisive role in the efficacy of ICI-based immunotherapy (10, 11). Besides, the activation of type I interferon (IFN-I) signaling contributes significantly to the expression of PD-L1 on these APCs (12). To be specific, upon sensing of DNA from dead cancer cells, the Cyclic GMP-AMP Synthase (cGAS)-Stimulator of Interferon Genes (STING)-IFN-I pathway within APCs or tumor cells can be activated, thus upregulating the Janus tyrosine kinase (JAK)-Signal Transducer and Activator of Transcription (STAT) signaling, and then improving PD-L1 on the surface of both tumor and host cells (13, 14). Therefore, identifying and targeting the crucial molecules that attenuate the STING-IFN-I pathway in the HNSCC microenvironment has become an important strategy to restore IFN-I signaling and improve the response rates of immunotherapy.

STING is a crucial pattern recognition receptor (PRR) for the activation of the IFN-I pathway, which promotes the phosphorylation of TANK-binding kinase 1 (TBK1), and in turn upregulates the level of phosphorylated interferon regulatory transcription factor 3 (p-IRF3), thereby activating the IFN-I pathway (15–18).

Previous studies have shown that SRY-box transcription factor 2 (SOX2) can promote the autophagic degradation of STING in HNSCC (19), leading to the inhibition of the IFN-I pathway and tumor immune evasion. Of note, another study revealed that upon human immunodeficiency virus (HIV) infection, NOD-like receptor family member X1 (NLRX1) can interfere with the interaction between STING and TBK1, resulting in the downregulation of the IFN-I pathway and a consequent negative regulation of antiviral innate immune responses (20). Further, our group’s prior research reported that Human Papilloma Virus 16 (HPV16) E7 can bind to NLRX1, mediating the autophagic degradation of STING and the inhibition of the IFN-I signaling pathway, thereby reducing tumor immunogenicity. This NLRX1-mediated autophagic mechanism can be targeted to restore immunogenicity of HPV16 positive HNSCC (21). However, the interaction between NLRX1 and the STING-IFN-I pathway in HPV negative HNSCC remains undetermined.

Prompted by the critical role of the STING-IFN-I pathway in anti-tumor immunity and the limited efficacy of ICIs in HNSCC, we investigated the potential association between NLRX1 and STING in tumor microenvironment and their function in the immune evasion. Based on our assays, we confirmed a significant negative correlation between NLRX1 and STING expression, as well as an inverse relationship between tumor intrinsic NLRX1 and PD-L1 levels expressed on APCs in HNSCC. Importantly, knockdown of NLRX1 increased expression of PD-L1 on APCs and enhanced the efficacy of PD-L1 therapy, providing a promising strategy to overcome ICI resistance in HNSCC.

## 2 Materials and methods

### 2.1 Clinical specimens and tissue microarrays

Patients were included into our study after receiving their consent before operation. A set of 58 HNSCC tissues were acquired from the West China Hospital of Stomatology for generating individual tissue microarray (TMA). The TMA was stained with NLRX1 (LS-C115894, LSBio, 1:200) and STING (13647, Cell Signaling Technology, 1:200) separately, the survival and relapse curves were depicted, and scores were computed to assess the relative expression levels. Secondary antibodies are goat anti-rabbit antibody (GK600505, Gene Technology, Shanghai). The scores were determined by multiplying the score of positively stained area by the intensity score (Score of positive area: <20%, 1; 20-50%, 2; 50-80%, 3; >80%, 4; Score of intensity: light, 1; medium: 2; strong: 3).

### 2.2 Immunohistochemistry

Tissue sections were deparaffinized following two times of wash in Dimethylbenzene, and treated for sodium citrate antigen retrieval for 4 minutes. After staining with primary antibody overnight, the sections were washed twice and treated with secondary antibody for 1h at room temperature. The 3, 3′-Diaminobenzidine (DAB) chromogenic kit (ZLI-9017, ZSGB-Bio) was applied to label the positive area. Immunochemistry (IHC) was performed with NLRX1(LS-C115894, LSBio) and STING (13647, Cell Signaling Technology). The secondary antibody employed in this experiment was a goat anti-rabbit antibody (ZB-2301, ZSGB-Bio). Blinding of two independent pathologists to the grouping during the evaluation of IHC results was executed.

### 2.3 Cell culture

HSC-3 (RRID: CVCL_1288), CAL-27 (RRID: CVCL_1107), CAL-33 (RRID: CVCL_1108), UM1 (RRID: CVCL_VH00) and SCC7 cells (RRID: CVCL_V412) were all provided by the State Key Laboratory of Oral Diseases of Sichuan University. RAW264.7 (RRID: CVCL_0493) was purchased from Fuheng Biology. All cell lines were tested negative for mycoplasma contamination. All cell lines were cultured in DMEM (Gibco), supplemented with 10% FBS and 1% penicillin-streptomycin (10378016, Gibco). Cell lines were all incubated in a humid atmosphere at 37 °C with 5% carbon dioxide (CO_2_).

### 2.4 Lentiviral transduction and plasmid transfection

Human and murine NLRX1-overexpression lentivirus was constructed and purchased from Genechem, human and murine NLRX1-knockdown lentivirus was constructed by Genepharma. HSC-3 were infected by NLRX1-overexpression lentivirus, CAL-27 were infected by both NLRX1-overexpression and NLRX1-knockdown lentivirus. SCC7 was transduced with Nlrx1-knockdown lentivirus. Empty vector virus was simultaneously utilized to infect according cells as negative controls. The screening concentration of puromycin was determined as 2 μg/mL for HSC-3, CAL-27, and 5 μg/mL for SCC7. After successful generation of the stable cells, immunoblotting was conducted for the confirmation. All infected cells were maintained with puromycin in the following experiments.

Human pcDNA3.1 STING–HA plasmids were kindly provided by Dr. Yu Leo Lei from the University of Texas MD Anderson Cancer Center. The transfection of pcDNA3.1 STING–HA was performed with 1 μg/ml plasmid and 2 times volume of Lipofectamine 2000 (11668019, ThermoFisher Scientific).

### 2.5 Cell proliferation and colony formation assay

To investigate the difference of cell proliferation potential, 1500 cells of SCC7 empty vector (EV)/Nlrx1^OE^ groups were inoculated into 96 well plates at day 0 respectively, and treated with cell counting Kit-8 (Dojindo, Japan) at 1:10 ratio for 2h before measurement of the absorbance starting from day 1 to day 4 at every 24h interval. As for colony formation assay, 300 cells of SCC7 EV/Nlrx1^OE^ sets were seeded into 6 well plates until the successful colony formation, and fixed in 4% paraformaldehyde followed by crystal violet staining for 15 minutes, images were then captured to reveal the cell number’s difference.

### 2.6 Animal experiments

All animal experiments were approved by the Ethics Committee of West China Hospital of Stomatology Sichuan University (WCHSIRB-D-2023-022). For all experimental procedures, mice were anesthetized with 4% isoflurane to ensure minimal discomfort and stress during handling and interventions. Six-week-old female C3H/HeN mice were purchased from GemPharmatech Co., Ltd. And 1×10^5^ SCC7 EV and Nlrx1^OE^ or Nlrx1^KD^ cells were injected into the right flank of C3H/HeN mice or NOD-SCID mice to construct subcutaneous tumor models. After tumor transplantation, the mice were randomly and equally divided into according groups, based on the sample size calculation; then, InVivoMab anti-mouse PD-L1 (BE0101, BioXcell) was dosing at 10 mg/kg per two days via intraperitoneal injection. Tumor volumes were measured regularly after tumor formation and were calculated using the formula 1/2 (length × width^2^). At the experimental endpoint, mice were humanely euthanized by overdose of CO_2_. Following CO_2_ euthanasia, complete cessation of respiration and heartbeat was confirmed to ensure death. The tumors, tumor draining lymph nodes (TDLNs), and spleens were obtained for further study.

### 2.7 RNA sequencing

Total RNA of HSC-3 EV/NLRX1^OE^ cells were collected with TRIzol reagent, and submitted to oebiotech (Shanghai, China) for RNA-seq sequencing, Gene Set Enrichment Analysis (GSEA) was then conducted to explore the significantly altered signaling pathway.

### 2.8 Immunoblotting

As for immunoblotting, RIPA buffer (P0013B, Beyotime Biotechnology) supplemented with protease and phosphatase inhibitor cocktail (78442, ThermoFisher Scientific) was applied to lyse and obtain the whole cell lysates on ice. Primary immunoblotting antibodies were described as follows: NLRX1(LS-C115894, LSBio), STING (13647, Cell Signaling Technology), phospho-TBK1 (5483, Cell Signaling Technology), TBK1 (3504, Cell Signaling Technology), PD-L1 (13684, Cell Signaling Technology), GAPDH (380626, ZSGB-Bio), α-tubulin (R23452, ZSGB-Bio); all these antibodies were diluted at 1:1000. Secondary antibodies utilized is goat anti-rabbit (ZB2301, ZSGB-Bio) and goat anti-mouse (ZB2305, ZSGB-Bio) at 1: 3000.

### 2.9 Quantitative real-time PCR

Total RNAs of cell lines or specimens were all extracted by RNA isolation kit (RE-03111, FOREGENE). The concentration of RNA was measured by the Nanodrop Spectrophotometer (ThermoFisher Scientific). cDNA was achieved after reverse transcribed of RNAs using PrimeScript™ RT reagent Kit (RR037B, Takara). The primers were produced by Sangon biotech, and the involved primer sequences were demonstrated in **Supplementary Table 1**. cDNA was 1:10 diluted and mixed together with 8 μl of SYBRgreen mix and 2 μl of primer mixture, forming a 15 μl mix and subjected to reaction in the Q5 instrument (ThermoFisher Scientific). The expression level of each transcript was calculated by using 2-ΔΔct algorithm.

### 2.10 Immunofluorescence

For immunofluorescence (IF) staining, paraffin embedded tissue sections were deparaffinized and rehydrated, then immersed in citrate antigen retrieval solution and subjected to high-pressure antigen retrieval for 4 minutes. After cooling to room temperature, sections were blocked with 5% BSA for 30 minutes. Primary antibodies, including CD68 (IR60961, DAKO) and PD-L1 (13684, CST, 1:200), or CD11c (ab52632, Abcam, 1:200), were incubated on the sections overnight at 4 °C. Subsequently, donkey anti-rabbit fluorescent secondary antibody (711-605-152, Jackson Immuno Research, 1:100) and donkey anti-mouse fluorescent secondary antibody (715-545-150, Jackson Immuno Research, 1:100) were co-incubated with the tissue sections at 37 °C for 90 minutes. Sections were then mounted with DAPI containing anti-fade mounting medium (H-1200, Vector Laboratories). Slides were scanned using an automated whole slide scanner (Aperio Versa, Leica).

### 2.11 Isolation of lymphocytes from TDLNs and spleen

Mouse TDLNs and spleens were harvested and immediately placed in RPMI medium. Single cell suspensions were prepared by passing the tissues through a 70 µm cell strainer. Splenocytes required red blood cell lysis using Red Blood Cell Lysis Buffer (BL503A, Biosharp). Subsequently, lymphocytes were isolated from the cell suspensions by density gradient centrifugation using Ficoll (17144002, Cytiva). Isolated lymphocytes were washed with RPMI medium and cryopreserved at -80 °C until further use.

### 2.12 Co-culture of RAW264.7 and SCC7 cells

RAW264.7 cells were stimulated with 20 ng/ml IFN-γ (315-05-100UG, PeproTech) and100 ng/ml LPS (L3129-10MG, MERCK) for 24 hours to induce M1 polarization. These polarized RAW264.7 cells were then directly co-cultured with adherent SCC7 cells (previously stimulated with cGAMP) for 48 hours. Subsequently, suspended RAW264.7 cells from the co-culture supernatant were collected for flow cytometry analysis.

### 2.13 Flow cytometry

Lymphocytes were harvested from TDLNs and spleens. Cells were stained with Zombie Aqua Fixable Viability Kit (423101, Biolegend) for 30 minutes, followed by Fc receptor blockade using TruStain FcX (101319, Biolegend) for 10 minutes. After washing, the flow antibody mixture was added and incubated for 30 minutes. Multi-fluorescent staining was performed by Alexa Fluor 700 anti-mouse CD45 (103128, Biolegend), PE/Cyanine7 anti-mouse CD68 (137016, Biolegend), Brilliant Violet 605 anti-mouse CD11c (117334, Biolegend), Brilliant Violet 421 anti-mouse PD-L1 (124315, Biolegend). Then antibodies were discarded and the samples were then washed followed by PBS resuspend. All stained samples were subjected to Beckman Coulter CyAn ADP flow cytometer for detection. Data were processed and displayed by FlowJo version 10 software and the according gating strategies were revealed.RAW264.7 cells were stained with Zombie Aqua (423101, Biolegend), PE/Cyanine7 anti-mouse CD86 (Biolegend, 105013), Brilliant Violet 421 anti-mouse PD-L1 (124315, Biolegend) and FITC anti-Mouse F4/80 (123107, Biolegend). CAL-27 EV/NLRX1^KD^ cells were stained with Zombie Aqua (423101, Biolegend) and Brilliant Violet 421 anti-human CD274 (PD-L1) (329714, Biolegend).

### 2.14 Statistics

Two-tailed unpaired test was used to analyze two groups. As for the comparisons among three or more groups, one-way or two-way ANOVA analysis were individually employed for study with one factor or two factors; moreover, Sidak’s multiple comparisons test was exploited for further analysis between 2 random sets. The correlation between the expression of NLRX1 and STING was analyzed using Pearson correlation. Log-rank (Mantel-cox) test was exploited in the analysis of the correlation between NLRX1 and survival or relapse of HNSCC cohorts. All statistics and data presentations were completed by utilizing GraphPad Prism9.0.0.

## 3 Results

### 3.1 The expression of NLRX1 correlates with unfavorable patient outcome

**Figure 1:**
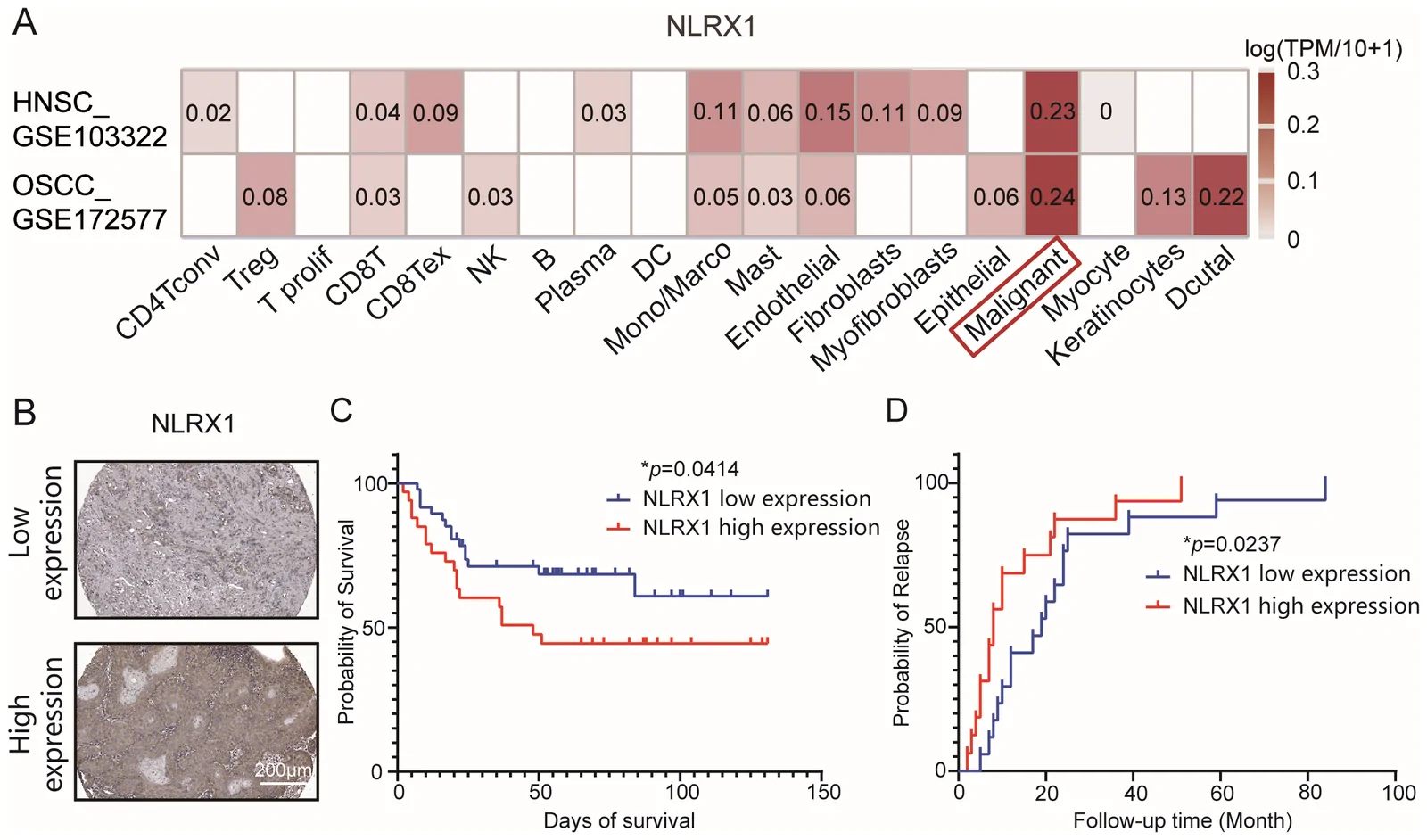
NLRX1 expression in HNSCC is associated with unfavorable prognosis. **(A)** The various distribution ratio of NLRX1 in different cell population by employing the single cell sequencing data revealed by TISCH. **(B)** TMA of HNSCC was stained with NLRX1 and scored. Representative images of IHC staining for NLRX1. **(C)** Kaplan-Meier survival curve was generated for 58 tissues and statistical analysis was performed using the Wilcoxon test. **(D)** 58 tissues were included in relapse curve and the comparison between 2 groups was conducted by the Log-rank test. (*p < 0.05).

Currently, most research regarding NLRX1 are concentrated on its role in antiviral signal transduction and triggering mitophagy (22–25); besides, our previous finding demonstrates that NLRX1 mediates the HPV initiated immune evasion of tumor, whereas none study has investigated the inherent function of NLRX1 in HNSCC. To gain initial insights into the cellular localization and potential function of NLRX1 within the HNSCC microenvironment, an analysis using Tumor immune single cell hub (TISCH) tool which integrates singlecell RNA-seq datasets was performed. The results demonstrated that NLRX1 may be predominantly expressed in malignant cells within the HNSCC tumor microenvironment (**Figure 1A**). This finding hints at potential association between NLRX1 and the malignant behavior of HNSCC.

Our subsequent analysis, TMA focused on determining the relationship between NLRX1 expression and HNSCC patient prognosis. Based on the scoring of IHC staining, 58 patients were stratified into NLRX1 low expression (IHC score ≤ 6) and NLRX1 high expression (IHC score > 6) groups (**Figure 1B**). Combining clinical survival information, Kaplan-Meier (K-M) survival and recurrence curves were generated and statistically compared. The results showed that patients in the NLRX1 high expression set exhibited significantly shorter post-operative overall survival (**Figure 1C**) and markedly increased rate of tumor recurrence compared to those in the NLRX1 low expression group (**Figure 1D**). These findings collectively indicate that NLRX1 is significantly associated with unfavorable clinical outcomes, including reduced patient survival and higher recurrence rates.

### 3.2 NLRX1 promotes tumor progression by modulating the immune microenvironment

Elucidating the mechanism by which NLRX1 contributes to malignant tumor progression is crucial for understanding its oncogenic role. Murine HNSCC cell line (SCC7) with Nlrx1 overexpression and its control set of EV were established by us to explore the regulatory role of NLRX1 on tumor growth both *in vitro* and *in vivo*.

**Figure 2:**
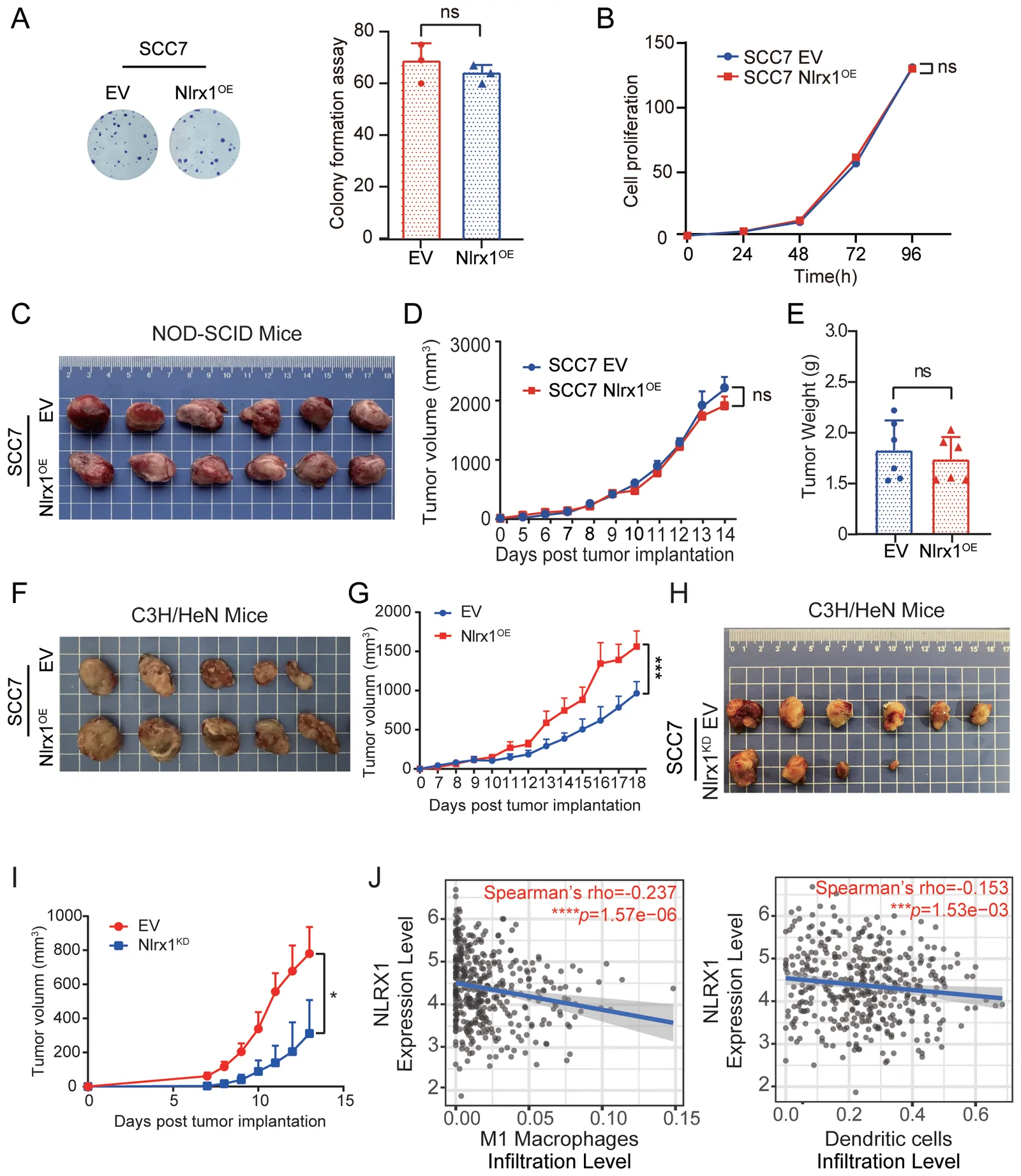
NLRX1 promotes tumor progression by predominantly modulating the immune microenvironment. **(A)** Colony formation assays were performed on SCC7 EV and Nlrx1^OE^ cells in three biological replicates. Representative images are shown in the left panel, and quantitative comparisons were made using a two-tailed unpaired t-test. **(B)** Proliferation differences between SCC7 EV and Nlrx1^OE^ cells were assessed using the CCK-8 assay. Data are shown as the mean of three independent experiments, and statistical significance was determined by two-way ANOVA. **(C)** 1 × 10^5^ SCC7 EV or Nlrx1^OE^ cells were subcutaneously inoculated into NOD-SCID mice. Representative image of tumors harvested from mice at the experimental endpoint was shown. **(D)** Tumor volumes were routinely measured and calculated to display the growth curve, comparison between indicated each 2 sets was made using two-way ANOVA. Representative results of two independent assays were shown. **(E)** Quantitative comparisons of tumor weight between SCC7 EV and Nlrx1^OE^ groups were made using a two-tailed unpaired t-test. **(F)** 1 × 10^5^ SCC7 EV or Nlrx1^OE^ cells were subcutaneously inoculated into C3H/HeN mice. Representative image of tumors harvested from mice at the experimental endpoint was shown. **(G)** Tumor volumes were routinely measured and calculated to display the growth curve, comparison between these 2 sets was made using two-way ANOVA. **(H)** 1 × 10^5^ SCC7 EV or Nlrx1^KD^ cells were subcutaneously inoculated into C3H/HeN mice. Representative image of tumors harvested from mice at the experimental endpoint was shown. **(I)** Tumor volumes were routinely measured and calculated to depict the growth curve, comparison between these 2 sets was performed using two-way ANOVA. Representative results of two independent assays were shown. **(J)** The transcriptional level of NLRX1 was negatively correlated with infiltration level of M1 macrophages and DCs in The Cancer Genome Atlas (TCGA) data of HNSCC by utilizing TIMER 2.0 with the ‘Purity Adjustment’ option enabled. (Quantifications present the mean ± SEM; *p < 0.05, ***p < 0.001, ****p < 0.000; ns, not significant.

The direct impact of Nlrx1 on the proliferative capacity of HNSCC cells was assessed through colony formation and CCK8 assays, utilizing SCC7 Nlrx1-overexpressed and its EV control. Both experiments consistently demonstrated that Nlrx1 cannot directly affect the proliferation of SCC7 cells *in vitro* (**Figures 2A, B**).

To elucidate whether the effect of Nlrx1 on tumor growth depends on adaptive immunity, we have compared the tumor growth of Nlrx1-overexpressing (Nlrx1^OE^) SCC7 cells with EV sets in both immunodeficient NOD-SCID mice and immunocompetent C3H/HeN mice. Notably, Nlrx1 overexpression exhibited none significant impact on tumor growth in NOD-SCID mice (**Figures 2C–E**). By contrast, in the syngeneic C3H/HeN model, Nlrx1^OE^ remarkably contributed to tumor progression and increased the endpoint tumor burden (**Figures 2F, G**). Moreover, Nlrx1 knockdown suppressed tumor progression in immunocompetent C3H/HeN mice (**Figures 2H, I**). Combined with our *in vitro* findings, these results suggest that Nlrx1 cannot directly regulate HNSCC cell proliferation but rather modulate tumor development by orchestrating the tumor immune microenvironment.

Tumor growth is substantially determined by the dynamics of its immune microenvironment. Next, a comprehensive correlation analysis between NLRX1 and the infiltration of immune subsets in HPV negative HNSCC was conducted by using the Tumor immune estimation resource 2.0 (TIMER 2.0) database. The results indicated a significant negative correlation between NLRX1 and M1 macrophages, dendritic cells (DCs) (**Figure 2J**). These observations further indicate that NLRX1 may exert an immunosuppressive function, thereby promoting the progression of HNSCC.

### 3.3 NLRX1 exerts regulatory effects on tumor immunity potentially through repressing IFN-I pathway

**Figure 3:**
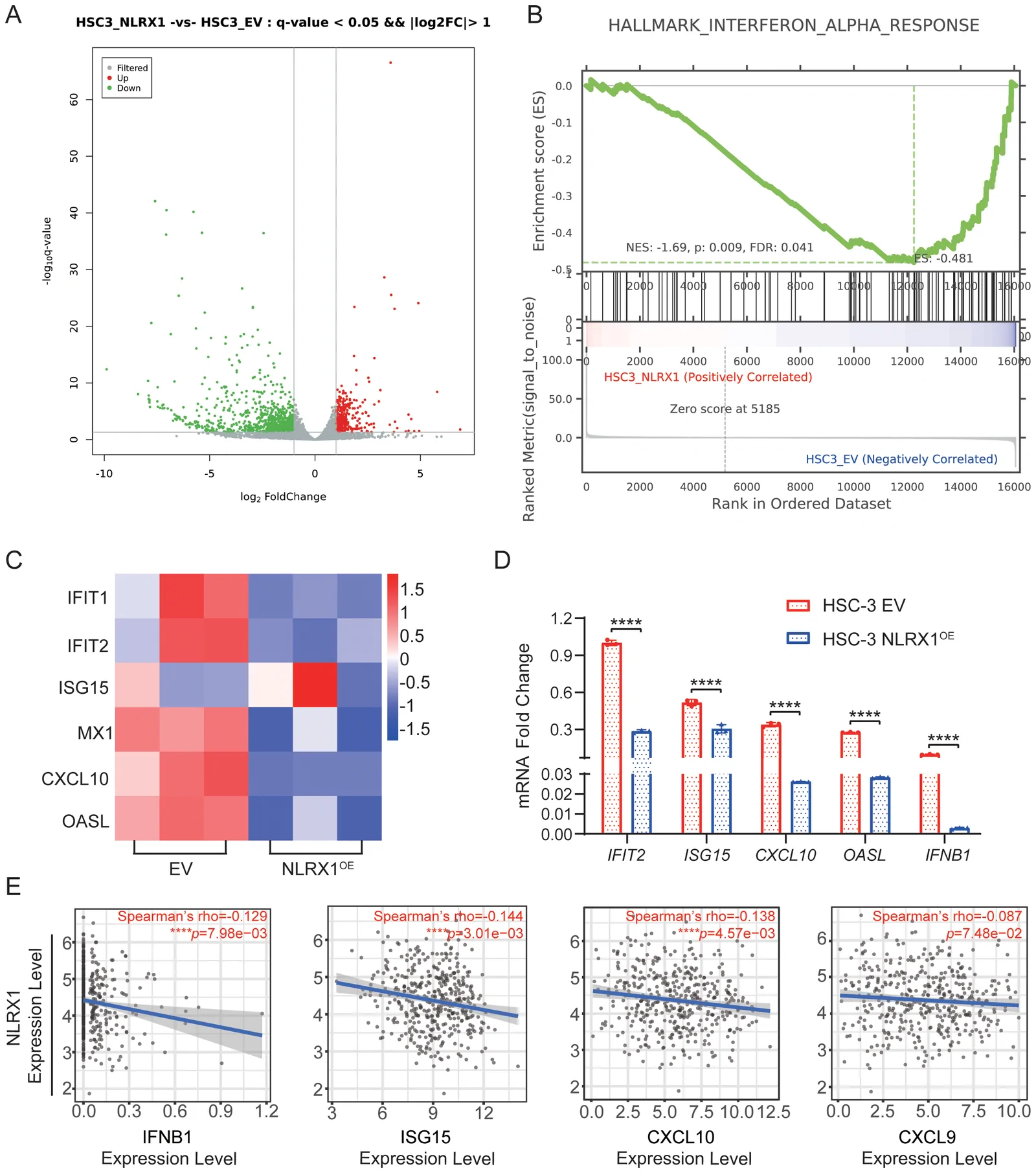
NLRX1 modulates tumor immunity by suppressing the IFN-I pathway. **(A)** Total RNAs of HSC-3 EV and NLRX1^OE^ cell lines were submitted to RNA sequencing. Volcano plot depicting the distribution of differentially expressed genes. **(B)** GSEA enrichment analysis of IFN-α pathway was displayed. NES=-1.69, *p* = 0.009, FDR = 0.041. **(C)** Heatmap illustrating the expression of IFN-I downstream genes based on RNA-seq data. **(D)** The mRNA expression levels of indicated genes were measured by q-PCR in HSC-3 EV and NLRX1^OE^ cells. (Each sample including three replicates; two-tailed unpaired t test was used). **(E)** The transcriptional level of NLRX1 was negatively correlated with expression of *IFNB1*, *ISG15, CXCL10* and *CLCL9* in TCGA data of HNSCC by utilizing TIMER 2.0 with the ‘Purity Adjustment’ option enabled. (Quantifications present the mean ± SEM; ****p < 0.0001).

To delineate the molecular pathways through which NLRX1 modulates tumor immunity, we performed transcriptome sequencing by utilizing HSC-3 NLRX1-overexpressing (NLRX1^OE^) and EV cells. Differential gene expression analysis identified 1349 significantly altered genes following NLRX1 overexpression, with 382 genes upregulated and 967 genes downregulated (**Figure 3A**). GSEA of the transcriptome data revealed that IFN-I pathway was significantly suppressed in NLRX1^OE^ cells (**Figure 3B**), supporting a negative regulatory role of NLRX1 on this pathway. Further, a heatmap of representative IFN-I downstream genes has indicated the marked decrease of these genes upon NLRX1 overexpression (**Figure 3C**).

To validate the transcriptomic finding, we performed quantitative real-time PCR (qPCR) on total RNA extracted from NLRX1^OE^ and control cells. As expected, significant reduction in expression levels of *IFNB1* and its downstream targets including *IFIT2*, *ISG15*, *CXCL10* and *OASL* were displayed (**Figure 3D**).

Consistently, analysis of HPV negative HNSCC data from the TIMER 2.0 database revealed that *NLRX1* expression is negatively correlated with the levels of *IFNB1* and its downstream targets (*ISG15* and *CXCL10*); meanwhile, though statistically non-significant, a trending negative correlation was observed for NLRX1 and *CXCL9* (**Figure 3E**). These integrated transcriptomic and bioinformatics analyses collectively demonstrate that NLRX1 can suppress the IFN-I signaling, thus potentially accounting for its regulatory role on tumor immunity.

### 3.4 NLRX1 negatively correlates with STING in HNSCC *in vitro*

**Figure 4:**
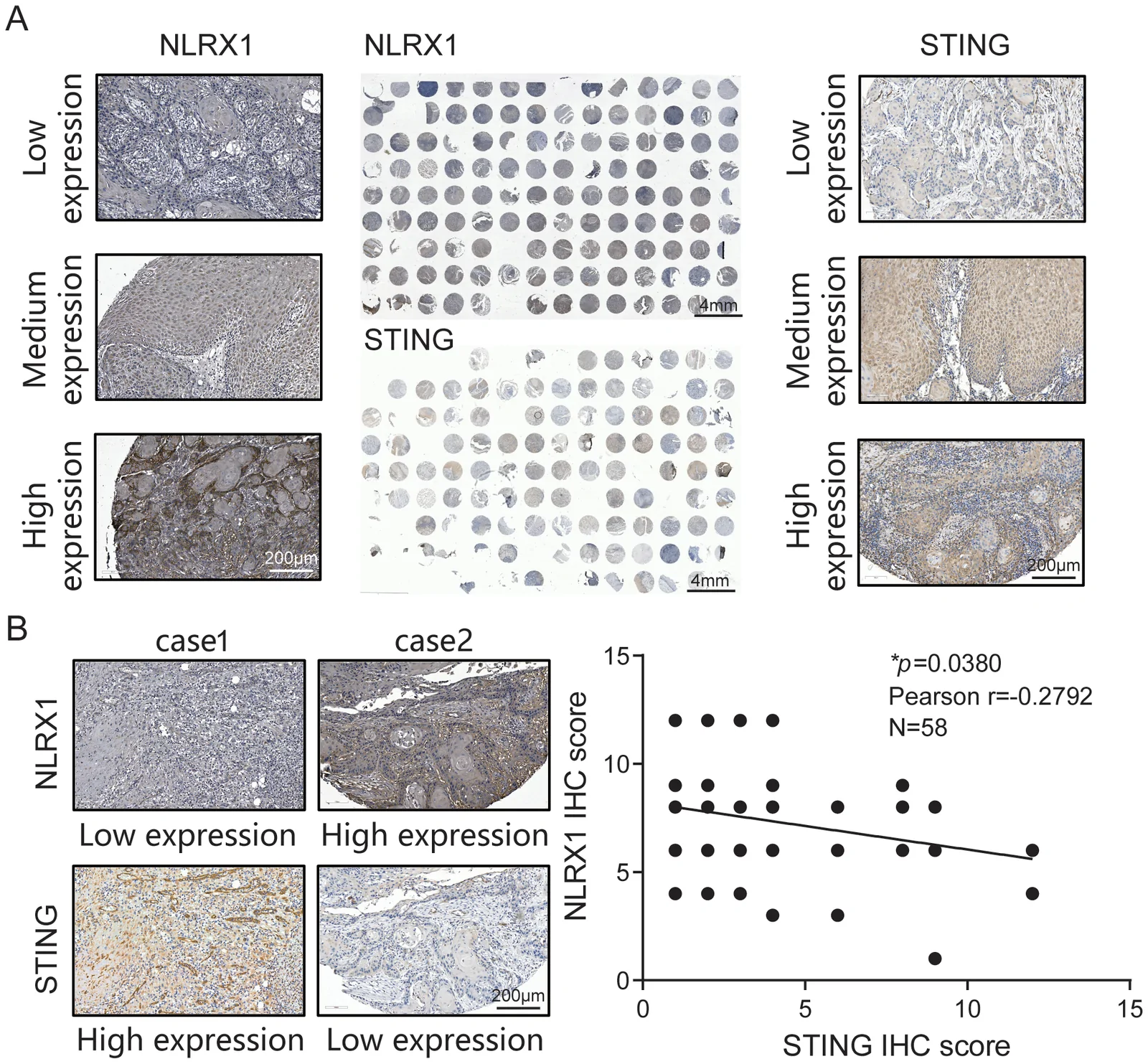
NLRX1 negatively correlates with STING expression. **(A)** Two TMAs were individually stained with NLRX1 or STING, and each available tissue was scored; staining of representative tumors are shown. **(B)** Pearson correlation analysis was conducted to depict the association between NLRX1 and STING (N=58 independent samples).

Given the above finding regarding the role of NLRX1 on modulating IFN-I pathway, the specific mechanism responsible for this remains largely uncharacterized. As STING is considered as a crucial pattern recognition receptor (PRR) for stimulating the IFN-I pathway (15), we investigated its potential relationship with NLRX1 in human HNSCC cohort. Of note, IHC staining of TMAs containing 58 HNSCC samples revealed a significantly negative correlation between the expression levels of NLRX1 and STING (**Figures 4A, B**). These findings collectively indicate that NLRX1 expression negatively correlates with STING in human HNSCC.

### 3.5 NLRX1 inhibits STING-mediated activation of the IFN-I Pathway *in vitro*

**Figure 5:**
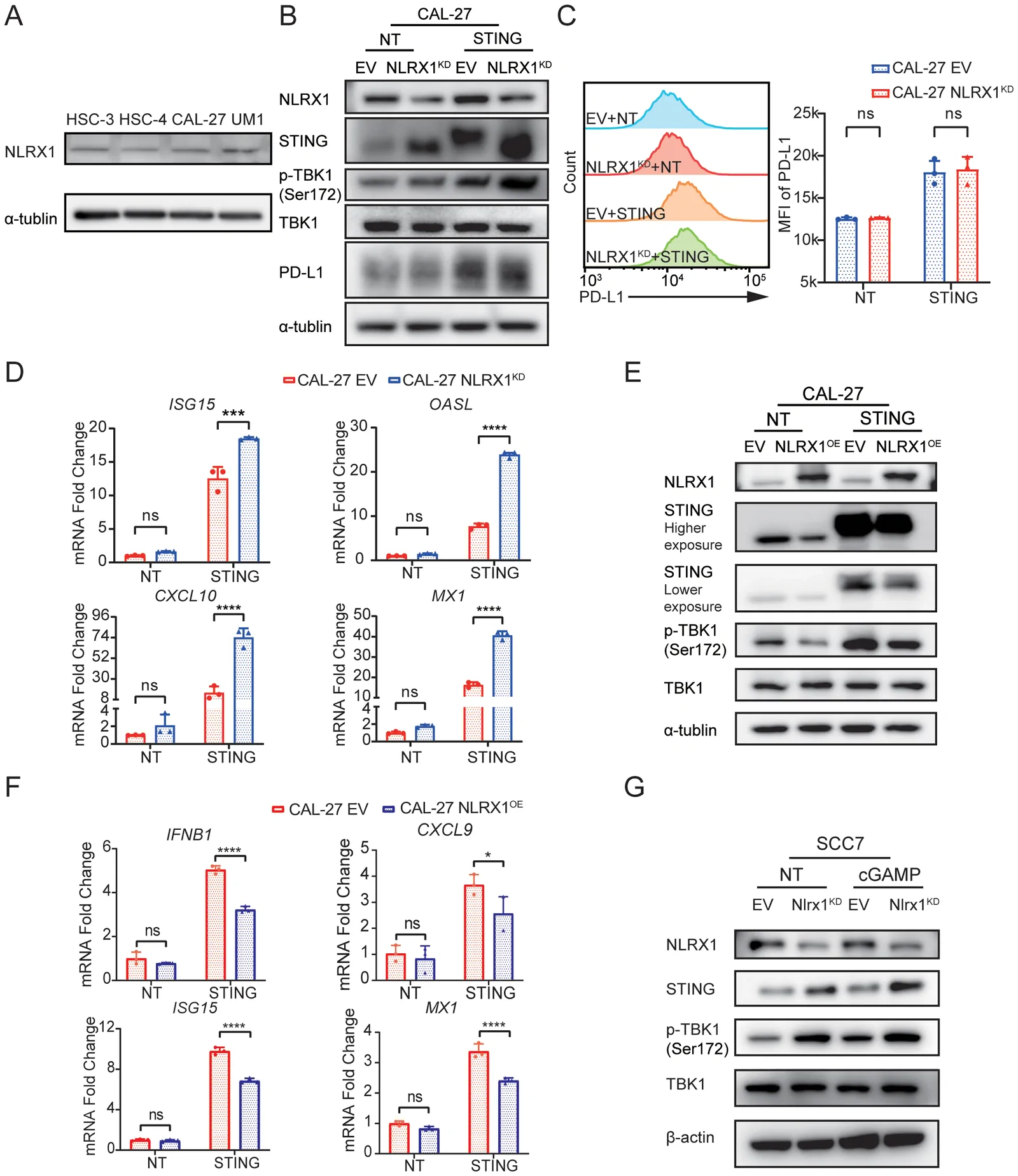
NLRX1 can negatively regulate the STING-IFN-I pathway. **(A)** Total proteins of HNSCC cell lines were subjected to immunoblotting for indicated proteins. Representative blots of three independent experiments are shown. **(B, D)** CAL-27 EV/NLRX1^KD^ and CAL-27 EV/NLRX1^OE^ cells were transfected with 1 μg/ml of pcDNA3.1 or STING plasmids by utilizing lipofectamine 2000 at 1:2 ratio for 16 hours; immunoblotting was conducted to confirm the activation of IFN-I pathway. Data indicates a representative graph of three independent biological replicates. **(C)** The transcripts of *IFNB1* and *OASL* were quantitated by q-PCR in CAL-27 EV/NLRX1^KD^ cells. **(E)** The transcripts of *IFNB1, CXCL9, ISG15* and *MX1* were quantitated by q-PCR in CAL-27 EV/NLRX1^OE^ cells. **(F)** SCC7 EV/NLRX1^KD^ cells were transfected with 2 μg/ml of pcDNA3.1 or cGAMP stimulation by utilizing lipofectamine 2000 at 1:2 ratio for 16 hours; immunoblotting was conducted to confirm the activation of IFN-I pathway. Two-way ANOVA analysis was applied for the comparisons of **(C, E)**. Representative data of **(A–E)** from three independent experiments was displayed. (Quantifications present as mean ± SEM, *p < 0.05, ***p < 0.001, ****p < 0.0001; ns, not significant).

To explore the potential effect of NLRX1 on STING at the cellular level, we first performed immunoblotting on a panel of HPV negative human HNSCC cell lines (HSC-3, UM1, CAL-27, and CAL-33). Among the HNSCC cell lines evaluated, HSC-3 and CAL-27 exhibited low endogenous NLRX1 expression, whereas UM1 and CAL-33 showed higher levels (**Figure 5A**). To determine if NLRX1 functionally suppresses STING and consequently downregulates the IFN-I pathway, NLRX1-overexpressing (NLRX1^OE^) and knockdown (NLRX1^KD^) cell lines of CAL-27 were established. As a result, NLRX1 knockdown robustly enhanced STING-induced IFN-I pathway activation. Specifically, upon STING plasmid transfection, NLRX1^KD^ cells showed an upregulation of phosphorylated TBK1 (p-TBK1) (**Figure 5B**). However, immunoblotting and flow cytometry showed NLRX1 cannot regulate the PD-L1 expression of tumor cells (**Figures 5B, C**). Concomitantly, q-PCR analysis revealed increased expression of IFN-I pathway genes including *ISG15*, *OASL*, *CXCL10* and *MX1* in NLRX1^KD^ cells following STING activation, confirming an augmented IFN-I response (**Figure 5D**). Conversely, NLRX1 overexpression in CAL-27 cells significantly suppressed STING-induced IFN-I activation, evidenced by the marked downregulation of p-TBK1 upon STING transfection (**Figure 5E**) and reduced mRNA levels of *IFNB1* and downstream IFN-I pathway genes including *CXCL9*, *ISG15*, and *MX1* (**Figure 5F**). These results underscore the inhibitory role of NLRX1 in STING-mediated IFN-I signaling in HNSCC. To further corroborate these findings, Nlrx1 was knocked down in SCC7 cells, followed by cGAMP stimulation to activate IFN-I signaling, resulted in higher p-TBK1 levels compared to empty vector controls (**Figure 5G**).

In all, these *in vitro* assays consistently establish a negative regulatory relationship between NLRX1 and STING expression, demonstrating that NLRX1 inhibits IFN-I pathway activation by targeting STING.

### 3.6 Targeting NLRX1 upregulates PD-L1 expression of APC and boosts immunotherapeutic efficacy in HNSCC

**Figure 6:**
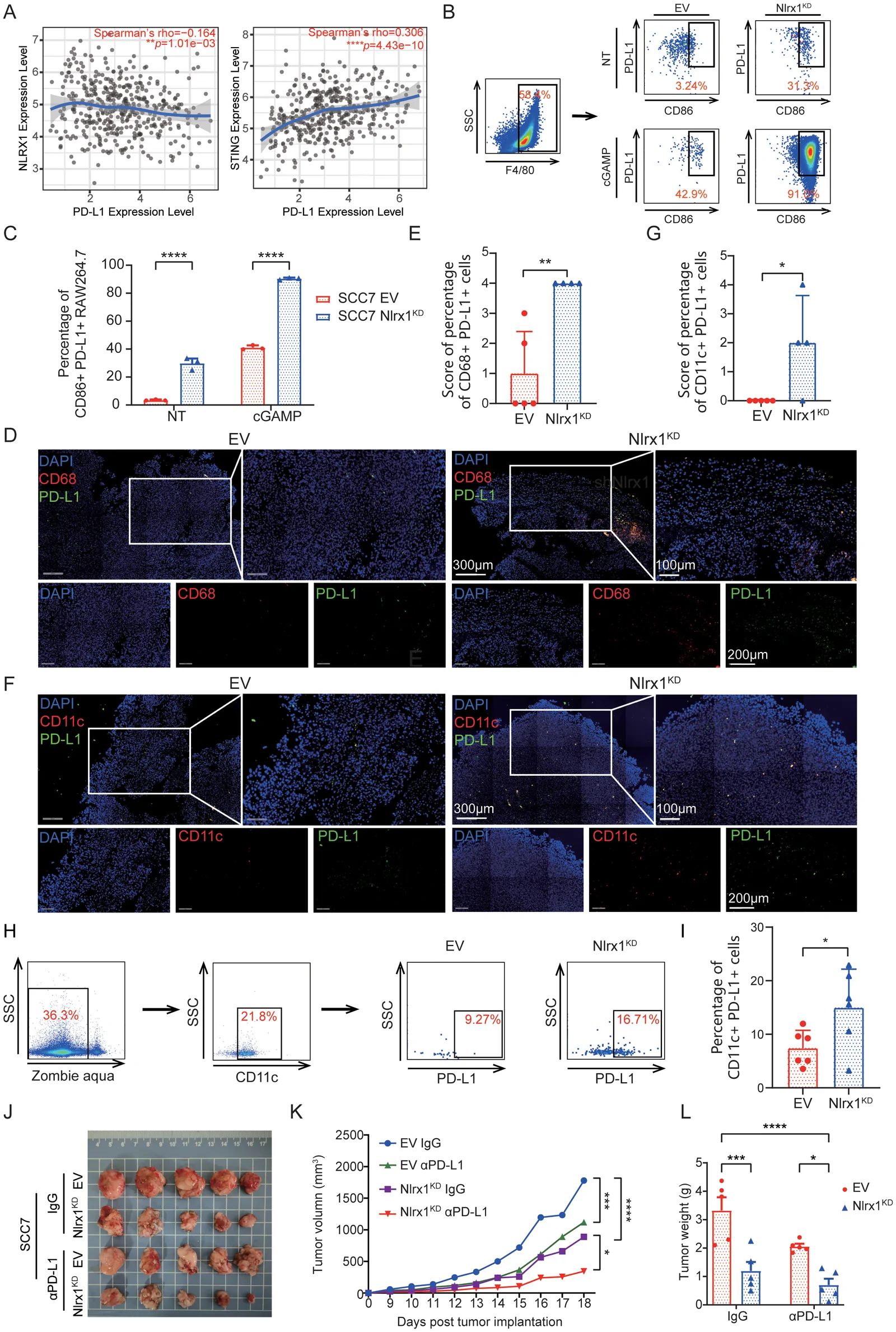
NLRX1 knockdown in HNSCC upregulates APC PD-L1 expression and enhances immunotherapeutic efficacy. **(A)** The transcriptional level of NLRX1 was negatively correlated with expression of PD-L1 and STING was positively correlated with expression of PD-L1 in TCGA data of HNSCC by utilizing TIMER 3.0 with the ‘Purity Adjustment’ option enabled. **(B)** Flow cytometric quantification of PD-L1+CD86+ RAW264.7 cell percentage. **(C)** Two-way ANOVA analysis was applied for the comparisons of percentage of PD-L1+CD86+ RAW264.7 cocultured with SCC7 EV/Nlrx1^KD^ cell transfected pcDNA3.1 or cGAMP. **(D)** Subcutaneous tumors of SCC7 EV or Nlrx1^KD^ cells were stained with CD68 and PD-L1 by IF. **(E)** Percentage of PDL1+ CD68+ cells of CD68+ cells of tumors were compared by two-tailed unpaired t-test. **(F)** Subcutaneous tumors of SCC7 EV or Nlrx1^KD^ cells were stained with CD11c and PD-L1 by IF. **(G)** Percentage of PDL1+ CD11c + cells of CD11c + cells of tumors were compared by two-tailed unpaired t-test. **(H)** Flow cytometric quantification of PD-L1+ CD11c+ TDLNs cell percentage. **(I)** Percentage of PDL1+ CD11c + cells of CD11c + cells of TDLNs cells were compared by two-tailed unpaired t-test. **(J)** 1 × 10^5^ SCC7 EV/Nlrx1^KD^ cells were subcutaneously injected into C3H/HeN mice, followed by intraperitoneal injections of IgG/anti-PD-L1 after tumor formation at day 11 on every other day. Representative image of tumors harvested from mice at the experimental endpoint was shown. **(K)** Tumor volumes were measured and computed to plot the curve. The experiments were performed twice and a representative curve was shown. (Two-way ANOVA was used for the comparison). **(L)** Quantitative comparisons of tumor weight between 4 groups were made using two-way ANOVA. (Quantifications present as mean ± SEM, *p < 0.05, **p < 0.01, ***p < 0.001, ****p < 0.0001).

As PD-L1 expression dictates the immunotherapy efficacy of HNSCC (6, 26), and IFN-I is a crucial regulator of the PD-L1 levels (27); thus, to assess the relationship between NLRX1, STING, and APCs’ PD-L1 expression in HPV negative HNSCC, bioinformatic study was primarily conducted. Our analysis based on TIMER 3.0 database initially revealed a negative correlation between *NLRX1* and *PD-L1* expression in HPV- HNSCC, contrasting with a significant positive correlation between *STING* and *PD-L1* (**Figure 6A**). This suggested a potential suppressive role of NLRX1 on PD-L1 levels within the tumor microenvironment.

To investigate how tumor cell-intrinsic NLRX1 affects macrophage phenotypes, M1-polarized RAW264.7 cells were co-cultured with either SCC7 Nlrx1-knockdown (NLRX1^KD^) or EV cells. RAW264.7 cells were subsequently analyzed for surface CD86 and PD-L1 expression by flow cytometry, and representative images were shown (**Figure 6B**). Flow cytometric analysis demonstrated that significant higher percentage of CD86+ PD-L1+ RAW264.7 cells can be observed in NLRX1 knockdown set under basal conditions. Moreover, cGAMP treatment has further increased the proportion of these CD86+ PD-L1+ macrophages in NLRX1 knockdown set compared with EV set (**Figure 6C**). Taken together, these *in vitro* co-culture findings indicate that tumor cell-intrinsic NLRX1 can potentially inhibit macrophage activation and restrain its antigen-presenting capacity, although its exact physiological contribution *in vivo* requires further study and cautious interpretation.

Further, we examined the impact of Nlrx1 knockdown on PD-L1 expression of APCs in HNSCC subcutaneous tumor model of **Figure 2F**. Immunofluorescence (IF) staining of tumor specimens for macrophage or DC with PD-L1, revealed that Nlrx1 knockdown significantly increased PD-L1 levels on both infiltrating macrophages and dendritic cells (**Figures 6D–G**), confirming the result of abovementioned co-culture assay *in vitro*. Concurrently, flow cytometry analysis of TDLNs demonstrated an elevated proportion of PD-L1+ DCs in the Nlrx1 knockdown group (**Figures 6H, I**). These *in vivo* findings indicate that Nlrx1 downregulates PD-L1 expression on APCs within the tumor microenvironment.

Given Nlrx1’s role in suppressing APC PD-L1 expression, we hypothesized that targeting Nlrx1 could enhance the efficacy of PD-L1 immunotherapy. Thus, *in vivo* study was performed to validate the synergistic effects of Nlrx1 knockdown with anti-PD-L1 treatment in a mouse HNSCC model. SCC7 EV and shNlrx1 cells were subcutaneously injected into C3H/HeN mice. Upon tumor establishment, mice received intraperitoneal injections of anti-PD-L1 mAb or control IgG (**Figure 6J**). While the SCC7 model showed slight responsiveness to PD-L1 treatment, Nlrx1 knock-down alone significantly delayed tumor growth. Remarkably, the combination of Nlrx1 knockdown and anti-PD-L1 mAb therapy contributes to the most robust tumor growth suppression (**Figure 6K**) and resulted in significantly reduced tumor weights (**Figure 6L**). Hence, our *in vivo* assays uncover that Nlrx1 knockdown can reverse the suppression of APC PD-L1 expression and significantly enhance the therapeutic efficacy of PD-L1 mAbs in HNSCC, positioning NLRX1 as a promising target to overcome αPD-L1 therapeutic resistance.

### 3.7 NLRX1 is negatively associated with APC PD-L1 expression in human HNSCC tissues

**Figure 7:**
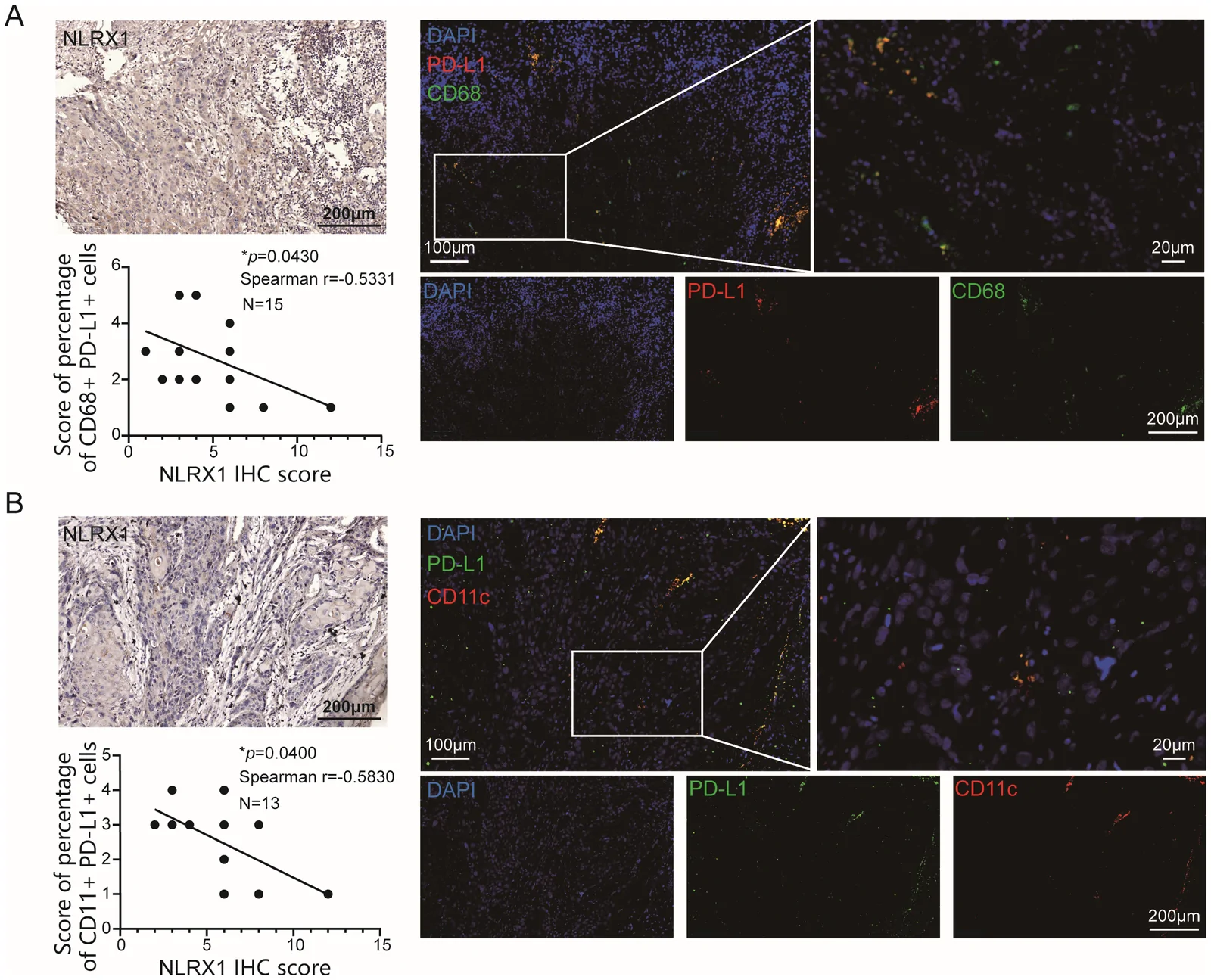
NLRX1 is negatively correlated with PD-L1 expression of APC subsets in HNSCC. **(A)** 15 human HNSCC samples was stained with NLRX1 and scored. The same samples were also co-stained with CD68 and PD-L1 by IF. Upper-left panel shows representative image of IHC staining for NLRX1. Right panel shows representative image of IF staining. Spearman correlation analysis was conducted to depict the association between NLRX1 IHC score and score of percentage of PD-L1+ CD68+cells. **(B)** 13 human HNSCC samples was stained with NLRX1 and scored. The same samples were also co-stained with CD11c and PD-L1 by IF. Upper-left panel shows representative image of IHC staining for NLRX1. Right panel shows representative image of IF staining. Spearman correlation analysis was conducted to depict the association between NLRX1 IHC score and score of percentage of PD-L1+ CD11c+ cells. (*p < 0.05).

Following our *in vivo* observations that Nlrx1 knockdown upregulates PD-L1 expression on APCs, we further investigated this in human HNSCC clinical samples. We assessed the relationship between NLRX1 expression in tumor cells and PD-L1 levels on distinct APC populations within the HNSCC tumor microenvironment using IF and IHC analyses. For macrophages, 15 HNSCC tissue samples were subjected to IF co-staining for CD68 and PD-L1. A similar approach was applied for DCs, where 13 HNSCC tissue samples were co-stained for CD11c and PD-L1, with PD-L1 expression on DCs estimated as the percentage of CD11c+PD-L1+ cells. Concurrently, NLRX1 expression within tumor cells of the same tissues was assessed by IHC. Our analyses demonstrated a significant negative correlation between tumor cell NLRX1 expression and the proportion of PD-L1+ macrophages (**Figure 7A**) and PD-L1+ DCs (**Figure 7B**). These findings consistently suggest an inverse relationship between NLRX1 in tumor cells and PD-L1 expression on APCs in HNSCC, underlying the resistance of NLRX1-highly expressed tumors to immunotherapy.

## 4 Discussion

HNSCC remains a formidable challenge in cancer treatment, with a significant proportion of patients experiencing limited benefits from ICIs therapies (3, 5). A major contributing factor to the low response rate is often attributed to insufficient infiltration and functional suppression of CD8+ T cells within the tumor microenvironment (TME) (28). Further, numerous studies have established a correlation between decreased PD-L1 expression in tumors and unsatisfactory immunotherapeutic outcomes (29, 30). Our study reveals that NLRX1, predominantly expressed in tumor cells, significantly promotes immune evasion by negatively regulating STING, thereby suppressing the IFN-I pathway and ultimately diminishing PD-L1 expression on APCs. More importantly, we demonstrate that targeting NLRX1 significantly increases APC PD-L1 expression upon IFN-I activation, thereby enhancing the efficacy of anti-PD-L1 therapy, presenting a potential strategy to overcome ICI resistance in HNSCC.

The role of NLRX1 in tumor immunity has been an area of emerging interest, though its precise mechanisms in cancer, particularly HNSCC, remain underexplored. While NLRX1 is primarily known for its involvement in antiviral signaling and modulating innate immune responses (22, 31), its contribution to tumor progression and immune evasion has been less characterized. For instance, previous work was chiefly concentrated on the inhibitory role of NLRX1 on STING-TBK1 interactions that downregulates IFN-I pathways in viral infections (20), and our prior research linked HPV16 to NLRX1-mediated autophagic degradation of STING, thus impairing tumor immunogenicity (21). Our current findings initially corroborates the function of NLRX1 in attenuating the IFN-I pathway in HPV negative HNSCC. We acknowledge that the negative correlation between NLRX1 and STING IHC scores in our HNSCC TMA was relatively weak, which was primarily attributable to the limited number of available cases (N = 58) during TMA processing. While this clinical trend achieved statistical significance and aligns with our mechanistic findings *in vitro*, future studies utilizing larger, independent clinical cohorts will be valuable to solidify this clinical association.

Multiple studies have implicated the pivotal role of autophagy in various aspects of tumor biology, including immune evasion (32–34). The process of autophagy involves the formation of autophagosomes, which engulf cellular components and deliver them to lysosomes for degradation (35). NLRX1 has been repeatedly reported to participate in the induction of autophagy (36, 37). Specifically, as the only NLR family member localized to the mitochondria, NLRX1 is a key player in mediating mitophagy (23, 24). In this study, we assume that NLRX1 may activate mitophagy, which in turn facilitates the degradation of STING. Future studies will delve deeper into the exact autophagic machinery involved in NLRX1-induced STING degradation, including the identification of specific adaptor protein that recruits STING to the autophagosome for targeted clearance.

The expression of PD-L1 on both tumor cells and APCs within the TME is a critical determinant of immune checkpoint blockade’s efficacy (10, 11). While tumor cell-intrinsic PD-L1 is recognized regarding its role in this scenario, emerging evidences have underscored the crucial function of PD-L1 on APCs in mediating effective anti-tumor responses and ICI sensitivity (10, 11). In line with that, our research, based on results from both bioinformatics analyses and direct IF staining of human HNSCC tissues, has revealed a negative correlation between tumor cell NLRX1 expression and PD-L1 levels on infiltrating macrophages and DCs, while demonstrated that NLRX1 cannot regulate tumor cell intrinsic PD-L1. We acknowledge that our human HNSCC tissue cohort is limited by a relatively small sample size (N = 13-15). Further validation in larger, independent patient cohorts will be valuable to consolidate these translational insights.

Further, the result from co-culture assay of SCC7 with macrophages has confirmed the finding. As an interpretation, we propose that this may be correlated with the relative abundance of APCs in TME of HNSCC. It should be noted that while our *in vitro* co-culture data suggest a potential cross-talk between tumor NLRX1 and macrophage PD-L1 expression, these observations are based on a cell culture model and warrant cautious interpretation regarding the complex *in vivo* tumor microenvironment. Further *in vivo* studies investigating the regulating role of NLRX1 on PD-L1 levels of APCs in HNSCC are warranted.

In summary, our study identifies NLRX1 as a critical mediator of immune evasion in HNSCC, contributing to resistance against immunotherapy. Our findings delineate a precise mechanism where NLRX1 negatively regulates STING, thereby suppressing the IFN-I pathway and subsequently downregulating PD-L1 expression on APCs. This orchestrated cascade ultimately attenuates efficacy of αPD-L1 and promotes HNSCC progression. Importantly, our preclinical data demonstrate that targeting NLRX1 can reverse these immunosuppressive effects, significantly enhancing the efficacy of PD-L1 blockade. This positions NLRX1 as a promising therapeutic target to suppress tumor growth and improve ICI’s response in HNSCC. In the future, we would be dedicated to developing small molecule inhibitors that specifically block NLRX1-mediated autophagy, thereby restoring the IFN-I pathway. The translational potential of these findings warrants further validation in larger clinical cohorts of HNSCC patients, paving the way for novel combination strategies to combat this aggressive malignancy.

## Data Availability

The datasets presented in this study can be found in online repositories. The names of the repository/repositories and accession number(s) can be found below: https://www.ncbi.nlm.nih.gov/, PRJNA1464131.
