# Supplementary material for: NLRX1 contributes to immunotherapeutic resistance of head and neck squamous cell carcinoma by suppressing type I interferon pathway
Source: Front Immunol. 2026 Aug 19;17:1890178. doi: 10.3389/fimmu.2026.1890178 (PMC13533696; doi:10.3389/fimmu.2026.1890178)
Supplement: Supplementary file 1 [file Table1.docx]

Supplementary Material

**Supplementary Table 1.** Primers sequences designed for the quantitive real-time PCR.

| Human | Forward: (5’-3’) | Reverse: (5’-3’) |
| --- | --- | --- |
| *IFNB1* | CATTACCTGAAGGCCAAGGA | CAATTGTCCAGTCCCAGAGG |
| *IFIT2* | TGCACTGCAACCATGAGTGA | GCCTCGTTTTGCCCTTTGAG |
| *CXCL9* | GTGGTGTTCTTTTCCTCTTGGG | ACAGCGACCCTTTCTCACTAC |
| *CXCL10* | CTCCAGTCTCAGCACCATGA | GCTCCCCTCTGGTTTTAAGG |
| *OASL* | GAATGGCACGGTTCTCAGGA | AAGAGCATAGAGAGGGGGCA |
| *ISGI5* | CTGAGAGGCAGCGAACTCAT | AGCATCTTCACCGTCAGGTC |
| *MX1* | CAATCAGCCTGCTGACATTG | TGTCTCCTGCCTCTGGATG |
| *GAPDH* | GAGTCAACGGATTTGGTCGT | TTGATTTTGGAGGGATCTCG |
